# Assessing the correlation between vastus medialis obliquus cross-sectional area and patellofemoral instability: a comparative magnetic resonance imaging study

**DOI:** 10.1007/s00402-025-06059-5

**Published:** 2025-09-16

**Authors:** Connor James Holmes, Diego Agustín Abelleyra Lastoria, Tobias Roberts, Vivian Ejindu, Claire Robertson, Caroline Hing

**Affiliations:** 1 School of Health and Medical Sciences, City St George’s University of London, London, UK; 2 St George’s University Hospitals NHS Foundation Trust, London, UK; 3 Croydon University Hospital, Thornton Heath, UK; 4 Fortius Clinic, London, UK

**Keywords:** VMO, Patellofemoral instability, MRI, Lateral patellar dislocation

## Abstract

**Background:**

This study investigated the relationship between the vastus medialis obliquus (VMO) cross-sectional area (CSA) and patellofemoral instability (PFI) in both primary and recurrent lateral patellar dislocations (LPD). Our secondary objective was to examine associations between VMO CSA and trochlear dysplasia, tibial tuberosity position, and patellar height in patients with PFI.

**Methods:**

Magnetic resonance imaging (MRI) radiographs were retrospectively analysed for 90 patients with primary acute LPD, 90 patients with recurrent LPD, and 56 patients without LPD (control). Measurements of the CSA ratio of the VMO to the whole thigh in three transverse slices were performed to calculate a mean ratio per patient. Additionally, tibial tubercle-trochlear groove (TT-TG) distance, patellar tilt angle (PTA), trochlear sulcus angle (TSA), and Insall-Salvati ratio (ISR) were measured as part of the Dejour Protocol.

**Results:**

The median CSA ratios in primary (0.04, standard deviation [SD]: 0.02) and recurrent (0.04, SD: 0.02) LPD patients were significantly lower than those in the control group (0.07, SD: 0.02) (*P* < 0.05). Compared with the primary LPD group, the recurrent LPD group presented significantly greater TT-TG distances (16.0, SD: 4.77 mm vs. 13.0, SD: 4.73 mm; *p* = 0.0101) and PTA (25, SD: 9.79 degrees vs. 19, SD: 15.76 degrees; *p* = 0.0071). There was no statistically significant correlation between any parameters of the Dejour Protocol and the VMO CSA ratio in patients with primary or recurrent dislocations (*P* > 0.05).

**Conclusion:**

Patients with both primary and recurrent LPD demonstrated smaller VMO bulk relative to the rest of the thigh compared with controls. These findings indicate an association between reduced VMO size and patellar dislocation; however, causality cannot be inferred from this cross-sectional analysis.

**Level of evidence:**

IV.

## Background

Patellofemoral instability (PFI) is a common disorder characterised by the symptomatic instability of the patella during normal movement. It predisposes patients to lateral patellar dislocations (LPD), which can lead to persistent pain, mobility issues, and weakness around the joint [1, 2]. The incidence of PFI is notably greater in children under 15 years of age [3].

Several anatomical pathologies have been implicated, including trochlear dysplasia and increased tibial tubercle-trochlear groove (TT-TG) distance [4]. Recurrent patellar dislocation rates range from 15 to 45% and is defined as the patella dislocating on more than one occasion [5, 6]. Trochlea dysplasia can be accurately assessed via either computed tomography (CT) or magnetic resonance imaging (MRI). The Dejour protocol, incorporating trochlear sulcus angle (TSA), TT-TG distance, patellar tilt angle (PTA), and Insall-Salvati Ratio (ISR), is widely used to evaluate these features and identify patients whose anatomy may predispose them to PFI, thereby guiding surgical decision-making [8].

The vastus medialis obliquus (VMO) provides countertraction medially to the patella during knee extension, restricting lateral patellar movement and stabilising the joint [7]. Previous studies suggest that morphological characteristics of the VMO, particularly its cross-sectional area (CSA), may influence PFI risk [2, 8–12]. However, a systematic review concluded that there is conflicting evidence regarding this matter [13].

In this context, this study aimed to investigate the association between VMO CSA and PFI in both primary and recurrent LPD. In parallel, we examined the relationships between VMO CSA and key anatomical parameters from the Dejour protocol – trochlear dysplasia, tibial tuberosity position, and patellar height – to provide a more comprehensive understanding of structural contributors to PFI [14–17]. We hypothesised that smaller VMO CSA would be associated with PFI in both primary and recurrent LPD patients.

## Methods

### Ethics

This study was registered as a service audit (AUDI003051) and evaluation with the Clinical Audit Department at St George's University Hospitals NHS Foundation Trust. Ethical approval was not required because this was a retrospective record review.

### Cohort and eligibility criteria

**Fig. 1:**
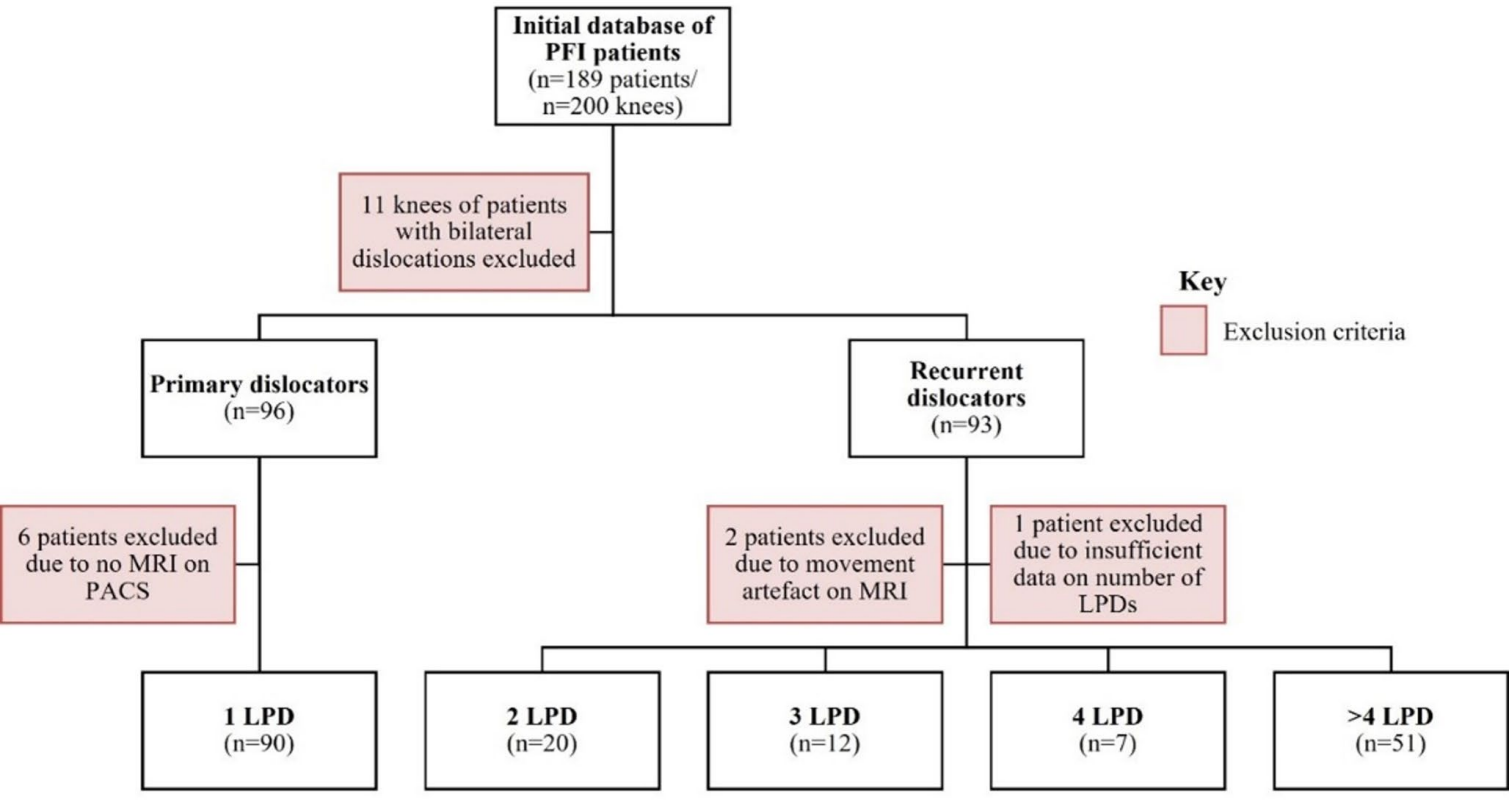
Flowchart displaying the application of inclusion and exclusion criteria to, and subsequent categorisation of, the study group dataset

**Fig. 2:**
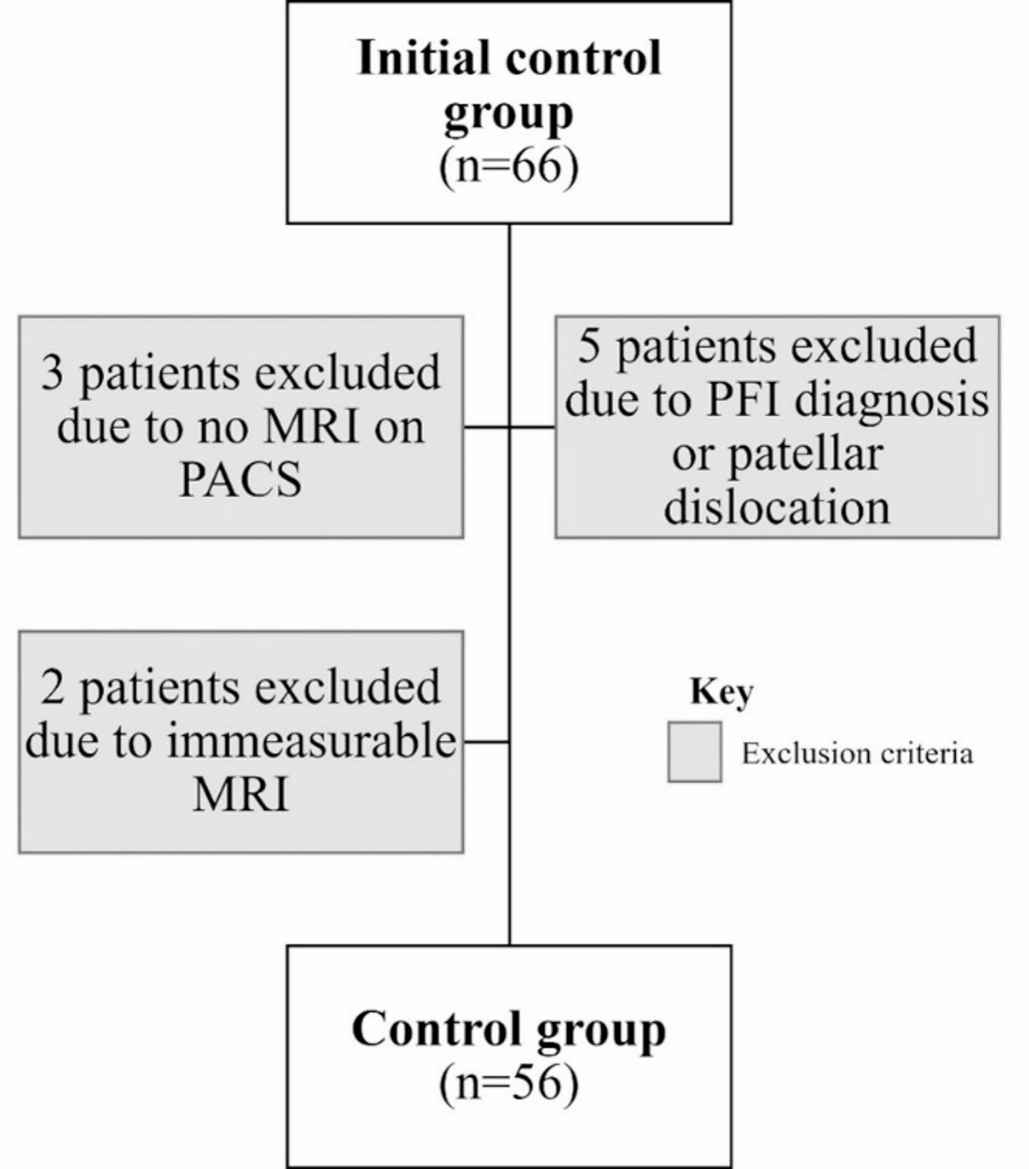
Flowchart displaying the application of inclusion and exclusion criteria for the formation of the final control group dataset

This was a single-centre retrospective study of patients treated in the PFI clinic for both primary and recurrent LPD, who consequently underwent MRI between January 2011 and December 2021. Clinic notes, imaging records, and attendance at our centre for any reason were reviewed for episodes of LPD, along with general practitioner (GP) summary care records. Primary LPD was defined as a patient with a single episode of LPD. Recurrent LPD was defined as any patient with more than one episode of patellar dislocation. In total, there were 189 patients (200 knees), of which 93 patients (100 knees) had primary LPD (Group 1), and 96 patients (100 knees) who experienced recurrent LPD (Group 2). Group 2 patients were further subdivided into those with two, three, four, or more than four dislocations (Fig. 1). A control group of 56 patients (Group 3) was identified from patients who had attended the knee clinic at our centre and underwent MRI for anterior cruciate ligament (ACL) rupture. They were screened for any mention of PFI or LPD in their medical history which would have excluded them from the study (Fig. 2). Controls were not matched to the LPD groups for age or sex. Data on body mass index (BMI) and physical activity level were not consistently available across all groups due to the retrospective nature of this study.

Among the 189 patients in the initial study group, 11 occurred twice in the dataset because they presented to the PFI clinic with bilateral dislocations, either concurrently or as separate incidents. These ‘duplicate’ patients were identified, and one of their knees each were randomly excluded, irrespective of whether the dislocations occurred concomitantly or separately. This was to avoid a repeated-measures problem, primarily due to the potential effect on demographic data, but also because the extent to which one dislocated knee may affect the patellofemoral joint on the other leg has not been quantified in the literature and may inadvertently skew the data [18].

Patients were excluded if they did not have appropriate imaging for analysis, if there was a lack of clarity from hospital records regarding the number of LPDs, or if the dislocation was medial. Patients in Group 3 (control) were excluded if there was documented evidence of PFI or dislocation.

### Vastus medialis obliquus-thigh cross-sectional area ratio

**Fig. 3:**
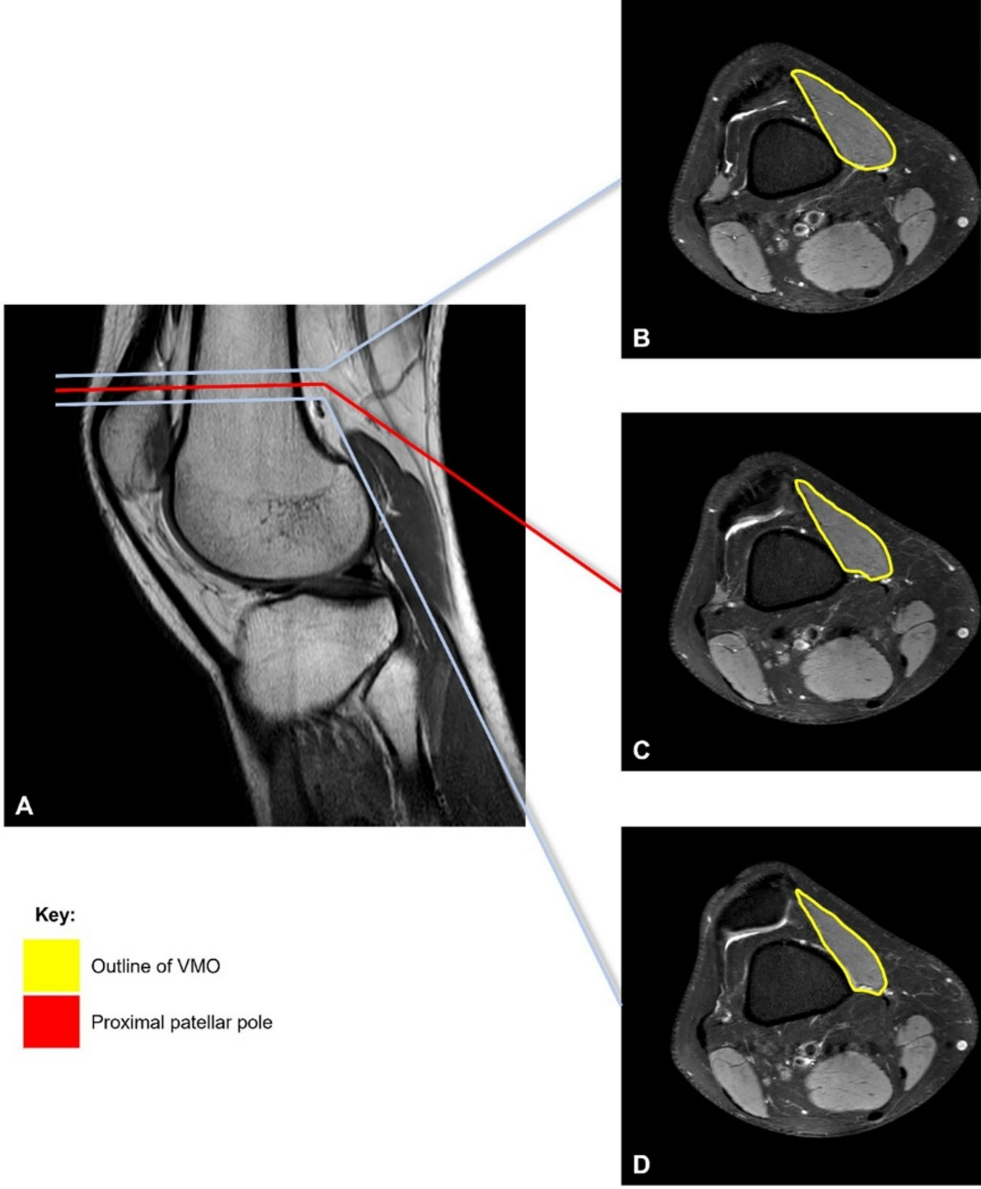
Measuring the cross-sectional area of the VMO. The sagittal slice corresponding to the midpoint of the posterior-most part of the patella in the transverse plane was located (**A**); the proximal patellar pole was identified and the corresponding axial slice (**C**), as well as the adjacent slices proximally (**B**) and distally (**D**) were measured

The method outlined by Balcarek et al. was followed. First, the proximal patellar pole (PPP) was identified in the central sagittal plane corresponding to the longitudinal patellar axis and correlated with the relevant axial slice [12]. Three slices were measured per knee: the axial (reference) slice located at the PPP, and the two adjacent slices, both immediately proximal and distal to the reference slice. The CSA of the VMO and the whole thigh were subsequently measured respectively on all three slices. The CSA ratio of the VMO was calculated as a percentage by dividing the CSA of the VMO by the CSA of the whole thigh (Fig. 3); the mean of the ratios for each slice was obtained to consolidate data for each patient.

In one case, the majority of the thigh extended over the borders of the MRI image and, as such, was excluded from the study. Measurements were taken via the picture archiving and communications system (PACS) workstation (Centricity, GE Healthcare, St. Gilles, United Kingdom), using the region of interest freehand measuring tool.

### Measurements of the Dejour protocol

The TSA is the angle between the medial and lateral femoral condyles forming the trochlear groove. Trochlear dysplasia can be diagnosed when the groove angle is > 145 degrees [19]. The TT-TG distance is a measurement of patellar translation, denoting the lateralisation of the tibial tuberosity in relation to the femoral trochlea [20]. The PTA is defined as the angle subtended by a line joining the medial and lateral edges of the patella and the horizontal edge [21]. PTA measurements were collected across the cartilage surface. The ISR is a measurement of patellar height and is the ratio of the length of the patellar tendon to the patella length. Compared with other calculations of patellar height, the ISR is more reliable and reproducible across both CT and MRI radiographs [22]. The normal and abnormal thresholds for each of the four measurements are summarised in Table 1.

**Table 1:** The thresholds of the Dejour protocol

Parameter	Normal threshold	Abnormal threshold
Trochlear sulcus angle [19]	< 145 degrees	> 145 degrees
Tibial tubercle-trochlear groove distance [16]	< 15 millimetres	> 20 millimetres
Patellar tilt angle [23]	< 15 degrees	> 20 degrees
Insall-Salvati ratio [24]	0.74–1.5	> 1.5

### Reliability of MRI assessment

MRI measurements were taken by a single observer. The measurements were repeated at six weeks to calculate intra-observer reliability. Images were also reviewed by a second observer, to calculate inter-observer reliability.

### Statistical analysis

Owing to the positive skew of all variables, confirmed by histogram analysis of the data, the parameters are presented as medians and standard deviations. The CSA ratio, TT-TG distance, ISR, PTA, and TSA for each group were assessed using the Kruskal-Wallis test. Additionally, the intraclass correlation coefficient was calculated using Pearson’s correlation coefficient for repeated measurements taken by two observers to test inter- and intra-observer reliability. Spearman’s rank correlation test was used to analyse if there was a monotonic association between the mean VMO CSA ratio and the TT-TG distance, ISR, PTA, and TSA in patients with a single or multiple patellar dislocations. The test statistic was denoted by ρ. The 95% confidence intervals of the correlation coefficient were estimated. The variance in the Spearman correlation was estimated by the method proposed by Fieller, Hartley, and Pearson. An α value < 0.05 was considered statistically significant. GraphPad Prism version 9.5.0 for Windows (GraphPad Software, San Diego, California USA) and SPSS version 29 (SPSS Inc, Armonk, New York, USA) were used to analyse the data.

## Results

### Demographics

The demographic data of all six groups are presented in Table 2. Six patients from group 1 and three patients from group 2 were excluded from the initial study group because of a lack of appropriate imaging data or insufficient clinical data. A flow diagram of the final study inclusion process is presented in Figs. 1 and 2.

Group 1 consisted of 26 men and 64 women. The median age was 23.8 (SD 9.54, range 9–61) years. Group 2 consisted of 27 men and 63 women. The median age was 24.7 (SD 8.98, range 11–62) years. Group 3 consisted of 37 men and 19 women. The median age was 29.6 (SD 8.49, range 16–63) years.

**Table 2:** The basic characteristics of the patients across the control and study groups

Group		Age, median ± SD (range), years	Sex, *n* (proportion)		Laterality, *n* (proportion)	
			Male	Female	Left	Right
Control (*n* = 56)		29.63 ± 8.49 (16–63)	37 (66%)	19 (34%)	26 (46%)	30 (54%)
Primary LPD (*n* = 90)		23.76 ± 9.54 (9–61)	26 (29%)	64 (71%)	61 (66%)	31 (34%)
Recurrent LPD (*n* = 90)	2 (*n* = 20)	25.50 ± 8.50 (12–43)	3 (15%)	17 (85%)	9 (45%)	11 (55%)
	3 (*n* = 12)	16.86 ± 7.84 (14–33)	7 (58%)	5 (42%)	11 (85%)	2 (15%)
	4 (*n* = 7)	27.88 ± 8.54 (11–37)	3 (43%)	4 (57%)	5 (71%)	2 (29%)
	> 4 (*n* = 51)	24.72 ± 9.51 (13–62)	14 (27%)	37 (73%)	32 (56%)	25 (44%)

### MRI assessment and analysis

The median VMO-thigh CSA ratio, TT-TG, PTA, TSA and ISR for each group are presented in Table 3. The median VMO-thigh CSA ratios of both Group 1 (4.03%) and Group 2 (4.14%) were significantly lower than that of Group 3 (6.71%) (*p* < 0.001). There was no statistically significant difference in the VMO CSA ratio between groups 1 and 2 (*p* = 0.575).

In addition, effect size estimates (η^2^) indicated that differences in VMO-thigh CSA ratio between groups represented a large effect, explaining a substantial proportion of the variance. Although TT-TG and PTA showed moderate effect sizes, these differences were not statistically significant and should therefore be interpreted cautiously. TSA and ISR demonstrated small effect sizes, consistent with their lack of significant group differences.

**Table 3:** Comparison of MRI measurements across control and study groups

		Cross-sectional area ratio, median (SD)	TT-TG distance, median (SD), mm	Patellar tilt angle of Laurin, median (SD), degrees	Trochlear sulcus angle, median (SD), degrees	Insall-Salvati ratio, median (SD)
Control (*n* = 56)		0.07 (SD 0.02)	–	–	–	–
Group 1: Primary LPD (*n* = 90)		0.04 (SD 0.02)	13.0 (SD 4.73)	19 (SD 15.76)	150 (SD 9.50)	1.40 (SD 0.23)
Group 2: Recurrent LPD (*n* = 90)	2 (*n* = 20)	0.03 (SD 0.01)	16.0 (SD 4.55)	21 (SD 7.38)	156 (SD 8.85)	1.36 (SD 0.33)
	3 (*n* = 12)	0.06 (SD 0.03)	15.5 (SD 4.58)	23 (SD 8.53)	151 (SD 11.9)	1.50 (SD 0.28)
	4 (*n* = 7)	0.04 (SD 0.02)	15.0 (SD 1.87)	28 (SD 15.16)	153 (SD 9.26)	1.50 (SD 0.26)
	> 4 (*n* = 51)	0.05 (SD 0.02)	17.0 (SD 5.00)	27 (SD 10.65)	154 (SD 14.26)	1.50 (SD 0.29)
	Overall (*n* = 90)	0.04 (SD 0.02)	16.0 (SD 4.77)	25 (SD 9.79)	154 (SD 12.73)	1.44 (SD 0.28)
P (comparison between all groups)		< 0.0001	0.0101	0.0071	0.4605	0.2682
η^2^ (comparison between all groups)		0.276	0.095	0.098	0.025	0.037

TT-TG and PTA were significantly greater in Group 2 than in Group 1 (*p* < 0.05). Differences in the TSA and ISR between groups 1 and 2 were not statistically significant. There was no statistically significant correlation between the TT-TG distance, ISR, PTA, or TSA and the VMO CSA ratio in patients with primary or recurrent dislocation (Table 4).

**Table 4:** Relationship between Dejour protocol parameters and VMO CSA ratio in patients with primary and recurrent patellar dislocation

Parameter	Primary dislocation (*n* = 68)	Recurrent dislocation (*n* = 72)
Tibial tubercle – tibial groove distance	ρ = 0.178, 95% CI: -0.075–0.498, *P* = 0.154	ρ = -0.27, 95% CI: -0.263–0.213, *P* = 0.825
Insall salvati ratio	ρ = -0.209, 95% CI: -0.444–0.053, *P* = 0.106	ρ = -0.047, 95% CI: -0.338–0.251, *P* = 0.752
Patella tilt angle	ρ = 0.050, 95% CI: -0.201–0.295, *P* = 0.691	ρ = -0.082, 95% CI: -0.316–0.161, *P* = 0.495
Trochlear sulcus angle	ρ = -0.135, 95% CI: -0.371–0.118, *P* = 0.281	ρ = 0.27, 95% CI: -0.212–0.264, *P* = 0.820

### Reliability of MRI measurements

There was excellent intra-observer reliability for MRI measurements (ICC: 0.94; 95% CI: 0.86–0.97; *p* < 0.0001), and excellent inter-observer reliability (ICC: 0.98; 95% CI: 0.96–0.99; *p* < 0.0001).

## Discussion

This study revealed that the VMO CSA ratio was significantly lower (*p* < 0.0001) in patients with lateral patellar dislocation than in those without. This finding is consistent with that of Shu et al., who reported similar results with a dataset of 75 knees in their study group [2]. Our results build upon the findings of Balcarek et al. and Liu et al., who demonstrated a downwards trend in the VMO CSA ratio in patients with recurrent LPD, although neither were able to establish statistical significance to corroborate this finding [12, 25]. Furthermore, Pompeo et al. reported that women with patellofemoral pain had a lower quadriceps muscle mass than did those without patellofemoral pain [26]. Across the study groups, the significantly increased results for TT-TG distance and PTA are in keeping with our expectations, considering that these factors have been previously demonstrated to be risk factors for PFI [5, 14, 28, 34].

We found that the VMO is significantly smaller in size in patients who have experienced LPD than in those who have not, suggesting that a smaller VMO relative to the rest of the thigh may lead to LPD. However, the VMO CSA ratios did not significantly differ between primary and recurrent patellar dislocation, suggesting that a low VMO-thigh CSA ratio is not causative of the development of recurrent patellofemoral instability. These findings also suggest that the development of recurrent PFI does not result in further VMO atrophy. This is in accordance with previous work, highlighting that PFI is a multifactorial phenomenon [2, 5, 14, 27, 28, 34]. The relationship between VMO size and PFI is likely bidirectional. While reduced VMO bulk may predispose patients to dislocation by providing less medial support, repeated dislocation and episodes of instability may also lead to muscle atrophy and weakening over time. This reciprocal effect creates a potential vicious cycle, making it difficult to distinguish whether a small VMO is a cause or a consequence of instability. Understanding this interplay is important for developing effective treatment strategies.

Trochlear osteology may be a more important factor than VMO CSA in predisposing patients to LPD recurrence. The TT-TG distance is a reliable measurement for the diagnosis of PFI, although some patients presenting with a TT-TG distance within normal limits but exhibiting signs of PFI may be further investigated by measuring the distance between the tibial tubercle and the posterior cruciate ligament [27, 28]. One study questioned the reliability of the Dejour protocol in classifying trochlear dysplasia [29]. However, it continues to be recommended as the most accurate categorisation method in clinical practice [30].

In addition to statistical significance, the large effect size we observed for the VMO-thigh CSA ratio further underscores its clinical relevance, suggesting that reduced VMO bulk accounts for a substantial proportion of the variance between patients with and without LPD. However, the relatively high standard deviations observed indicated considerable biological variability within the groups, which is plausible given individual differences in muscle morphology, physical activity levels, and potential compensatory mechanisms. While TT-TG distance and PTA showed moderate effect sizes, their lack of statistical significance indicates these factors may have a less consistent influence on PFI within this cohort. Similarly, small effect sizes and non-significant differences for TSA and ISR support the notion that these anatomical parameters play a limited role in the instability observed. Collectively, these findings highlight the importance of VMO morphology as a key factor in patellar stability and support its consideration in both diagnostic and therapeutic strategies.

Importantly, correlation analyses demonstrated very weak and non-significant relationships between VMO CSA ratio and the Dejour parameters. This negative finding highlights that, while the VMO size is clearly associated with patellar dislocation, it appears to operate independently of bony anatomical variations assessed by the Dejour protocol. This dissociation supports the multifactorial nature of PFI and suggests that soft tissue and bony factors may contribute through distinct mechanisms. Recent studies have highlighted the emerging value of dynamic assessment techniques for patellar tracking and tilt, which offer insights beyond those provided by the static anatomical measures evaluated in this study [31]. Moreover, strong correlations have been demonstrated between anatomical risk factors and dynamic patellar maltracking, reinforcing the complex interplay of factors underlying PFI [32]. Overall, these findings emphasise the importance of considering both muscular and anatomical assessments in the comprehensive evaluation and management of patients with PFI.

By measuring the VMO across multiple slices per patient, we were able to accurately replicate the three-dimensional morphology of this muscle. A separate study, which focused on the musculature of the shoulder, detailed the merits of measuring the CSA of muscles to simulate their three-dimensional structure as reliably accurate and clinically relevant. However, this was limited by being a cadaveric study, which hinders the in-vivo applicability of their results [33]. We included the whole thigh area in the calculation, rather than only the VMO area, to produce a ratio. This minimised inter-individual variability, indicating that this measurement methodology should be followed in further work assessing the CSA of other muscle compartments under MRI to maximise the reliability of the results.

Apart from one small in-vitro study, there is a limited understanding of the role of quadriceps training in PFI, and no studies have investigated whether the reduction in CSA is reversible [34]. The VMO, distinctly amongst the quadriceps muscle group, contributes to normal patellar kinematics during motion of the patellofemoral joint [35]. However, exercises cannot isolate the VMO. Electromyography data demonstrate less relevance of the motor recruitment of the VMO and more importance of the role of exercise on VMO architecture [36, 37]. In addition, previous cadaveric and in-vivo studies have shown that VMO weakness may play a role in dislocation [38–42]. A possible implication of our findings is that there is clinically relevant potential for targeted programmes strengthening the VMO to address PFI. Although this has been explored in previous studies, whether exercising the VMO leads to increased muscle power and decreased rates of LPD has yet to be explored [32]. Further research should aim to determine the most appropriate method for achieving this goal.

Our study was limited by the fact that the Dejour protocol was not implemented in the MRI scans of the control group as it is not indicated for ACL injury. Consequently, these findings are limited by the lack of comparisons with a healthy cohort. While the ACL-injured patients served as controls in this study, it is important to consider that ACL injury may influence quadriceps muscle morphology, potentially resulting in altered muscle CSA, particularly more proximally along the thigh [43, 44]. However, existing research, including an investigation of female athletes with ACL injuries, has shown no significant reduction in quadriceps strength following injury [45]. Nevertheless, it remains possible that ACL injury and the associated rehabilitation or disuse could have subtle effects on muscle bulk or quality, which may confound comparisons. The use of this cohort as a control group was driven by the challenges inherent in assembling a sufficiently large health control group in a retrospective study design.

In addition, variability in VMO CSA observed across all groups is likely influenced by multiple factors such as physical activity levels, body mass index (BMI), and genetic predispositions. Due to the retrospective nature of this study, detailed information on these parameters was not available for analysis, which limits the ability to fully account for their impact on muscle size and morphology. This variability was particularly noticeable in patients with substantial adipose tissue surrounding the femoral compartment, which may influence CSA measurements relative to total thigh size. Nonetheless, to preserve the representativeness of our sample and reflect real-world clinical populations, such individuals were not excluded from the analysis.

Another demographic consideration is the sex distribution differences between groups; the control group included a higher proportion of males compared to the predominantly female study groups. This is consistent with the known epidemiology of PFI, which disproportionately affects females, partly due to anatomical differences such as increased femoral anteversion and a higher prevalence of trochlear dysplasia [46–49]. While this demographic mismatch is a limitation, the groups reflect the populations encountered in clinical practice rather than a perfectly matched experimental sample.

Furthermore, a notable limitation of this study is the lack of control for patella alta, which can alter patellofemoral kinetics. Patella alta, defined by an ISR greater than 1.2, describes an abnormally superior position of the patella in the femoral trochlear groove [50, 51]. This superior displacement results in decreased patellar engagement within the trochlear during early knee flexion, increasing lateral patellofemoral joint stress and consequently elevating the risk of LPD [52, 53]. The variability in ISR within our cohort may, therefore, have influenced the risk profile for PFI and introduced heterogeneity to the data. Given that patella alta itself is a recognised independent risk factor for LPD, future studies should aim to stratify patients based on ISR to better elucidate its specific impact on VMO morphology and PFI.

These limitations highlight the complexity of interpreting VMO CSA measurements in the context of PFI. The interplay between muscle morphology, bony anatomy, demographic factors, and injury history underscores the multifactorial nature of this condition and the need for comprehensive prospective studies with carefully matched control populations and detailed clinical data, including physical activity levels, BMI, and patellar height metrics.

## Conclusion

A low VMO-to-thigh CSA ratio was significantly associated with lateral patellar dislocation, supporting its role in patellar stability. TT-TG distance and PTA, but not TSA or ISR, were significantly linked to recurrent dislocation. VMO CSA did not influence the risk of recurrent LPD, suggesting muscle atrophy may not worsen with repeated instability. These findings highlight the multifactorial nature of PFI. The control group consisted of patients investigated for ACL injuries.

## Data Availability

No datasets were generated or analysed during the current study.

## Abbreviations

VMO: Vastus medialis obliquus
CSA: Cross-sectional area
PFI: Patellofemoral instability
LPD: Lateral patellar dislocations
MRI: Magnetic resonance imaging
TT-TG: Tibial tubercle-trochlear groove
PTA: Patellar tilt angle
TSA: Trochlear sulcus angle
ISR: Insall-Salvati ratio
SD: Standard deviation
MPFL: Medial patellofemoral ligament
CT: Computed tomography
ACL: Anterior cruciate ligament
PPP: Proximal patellar pole
PACS: Picture archiving and communications system
